# Supplementary material for: Genuine selective caspase-2 inhibition with new irreversible small peptidomimetics
Source: Cell Death Dis. 2022 Nov 15;13(11):959. doi: 10.1038/s41419-022-05396-2 (PMC9666555; doi:10.1038/s41419-022-05396-2)
Supplement: Supplementary file 1 — supplementary information [file 41419_2022_5396_MOESM1_ESM.pdf]

Supplementary Information

Supplementary Figures

Figure S1

(A) Superposition of the NH / Hα regions of the NOESY (cyan) and TOCSY (magenta) spectra recorded on the LJ3a sample. The superimposition of the two spectra makes it possible to identify each amino acid by the TOCSY experiment and the sequential succession of these residues by the NOESY experiment. Sequential assignment is indicated by horizontal and vertical lines. The resonances are doubled for the positions P3 (Val 3) and P1 (Asp 1), then two sets of data can be identified, labeled in thin and in bold lettering and corresponding to two different isomers. (B) Table of chemical shifts ^{15}N and ^{1}H of the skeleton and side chains of the different residues of LJ3a. It is found that the chemical shift values are similar for the protons of the side chains and different for the ^{15}N, HN and Hα.

Figure S2

(A) Superposition of the NH / Hα regions of the NOESY (cyan) and TOCSY (magenta) spectra recorded on the LJ3b sample. The superimposition of the two spectra makes it possible to identify each amino acid by the TOCSY experiment and the sequential succession of these residues by the NOESY experiment. Sequential assignment is indicated by horizontal and vertical lines. The resonances are doubled for the positions P3 (Val 3) and P1 (Asp 1), then two sets of data can be identified, labeled in thin and in bold lettering and corresponding to two different isomers. (B) Table of chemical shifts ^{15}N and ^{1}H of the skeleton and side chains of the different residues of LJ3b. It is found that the chemical shift values are similar for the protons of the side chains and different for the ^{15}N, HN and Hα.

Figure S3

Effect of LJ3a on reversine-induced Mdm-2 cleavage. **A.** Proliferating human neural progenitor cells (H9 hESC-derived NPCs) were treated for 24h with 1µM reversine in the presence of the indicated concentrations of LJ3a. Then, cell extracts from triplicate wells were pooled and subjected to WB to detect full-length (FL) and cleaved (CL) forms of Mdm-2, as well as Casp2 and Casp3. A representative experiment is shown. **B.** Quantification of Mdm-2 cleavage inhibition. Mdm-2 Cl bands were quantified by densitometry analysis relatively to GADPH using panels in A and two independent repeats of this experiment. The histogram represents the means % inhibition (±SD) of Mdm-2 cleavage. * $P < 0.05$; ** $P < 0.01$; *** $P < 0.001$ (one-way Anova; n = 3).

Figure S4

Absence of Casp3 activation in HEK 293 cells transfected to overexpress SREBP2, S1P, PIDD1, RAIDD, and Casp2. **A.** HEK293 cells were prepared (5X10^5 per well) in 6-well plates. The next day, cells were transfected with the indicated plasmids (as in Figure 8). After 5h, cells were incubated in DMEM/F12 medium supplemented with DMSO (Veh.), LJ3a, or Δ2ME-TRP601 (10 μM) for 16h. Then, whole cell extracts (WCL) were prepared subjected to WB analysis to detect activated Casp3 using an antibody directed against cleaved Casp3 (Cell signaling #9661; diluted 1:1000). **B.** HEK293 cells were plated as in A. The next day, cells were transfected with the indicated plasmids. After 16 hrs, whole cell extracts (WCL) were prepared and Pro-Casp3 as well as Cleaved (Cl.) Casp3 were detected by WB using an antibody solution that includes two Casp3 antibodies (anti-proCasp3, Cell signaling # 9662) and (anti-cleaved Casp3, Cell signaling #9664), at 1:1000 ratio. Upper panel: HSP90 detection. Central panel: pro-Casp3/Cl.Casp3 detection, membrane exposed for 15 seconds. Lower panel: pro-Casp3/Cl.Casp3 detection, membrane exposed for 10 minutes. Optic density quantification (Image J; NIH, US) of WB bands for Pro-Casp3 and HSP90 showed no significant differences in the pro-Casp3/HSP90 ratio between empty vector and PIDDosome-transfected conditions.

Supplementary Tables

Suppl. Table 1

Comparison of the distances measured between protons on each 3D theoretical model of the 1R2R, 1S2R, 1R2S and 1S2S isomers and the volumes and distances derived from the NOESY experiment at 400 ms between these same protons. This comparison made it possible to identify the 2R or 2S isomer present in each sample and to conclude that LJ3a contains isomer 1(R,S)2R and that LJ3b contains isomer 1(R,S)2S. (*) indicates that two signals are superimposed.

Suppl. Table 2

Comparison of the distances measured between protons on each 3D theoretical model of the 1R2R, 1S2R, 1R2S and 1S2S isomers and the volumes and distances derived from the NOESY experiment at 100 ms between these same protons. This comparison made it possible to identify the 2R or 2S isomer present in each sample and to conclude that LJ3a contains isomer 1(R,S)2R and that LJ3b contains isomer 1(R,S)2S. (∅) indicates that no signal is detectable.

Suppl. Table 1

Comparison of the Measured distances on the model structures with the measured volumes and derived distances from the 400 ms NOESY spectra.

Measured distance on the model structures (Å)						Measured volumes and derived distances on 400 ms NOESY spectra				Result
Protons		1R2R	1S2R	1R2S	1S2S	LJ3a1	LJ3a2	LJ3b1	LJ3b2	
H21	H30	4.39	4.28	1.80	2.62	550018<br>4.43	*	820696<br>3.90	693644<br>4.00	LJ3b=1(R,S)2S
H21	H31	5.21	5.14	3.27	3.70	294592<br>4.92	354974<br>4.77	1050000<br>3.74	976086<br>3.74	LJ3b=1(R,S)2S
H22	H35	5.51	5.61	>6.0	>6.0	481831<br>4.04	481831<br>4.04	320176<br>4.06	320176<br>4.06	LJ3a=1(R,S)2R
H30	H46	4.89	4.87	4.00	4.08	110876<br>5.16	110876<br>5.16	96120<br>4.96	96120<br>4.96	LJ3b=1(R,S)2S
H31	H46	4.48	4.39	2.58	2.63	214238<br>4.63	214238<br>4.63	800000<br>3.49	800000<br>3.49	LJ3b=1(R,S)2S
H32	H37	4.21	4.07	4.39	4.23	520824<br>4.48	656653<br>4.31	346066<br>4.50	470582<br>4.28	LJ3a=1(R,S)2R
H33	H36	3.76	3.92	2.52	3.16	554349<br>4.43	801356<br>4.17	801016<br>3.91	1010000<br>3.76	LJ3b=1(R,S)2S
H34	H37	>6.0	5.69	5.55	4.30	239313<br>5.00	231267<br>5.13	113630<br>5.00	100462<br>5.50	LJ3a=1(R,S)2S
H37	H46	4.68	2.36	>6.0	5.96	89113<br>6.00	94410<br>5.96	65388<br>5.94	67752<br>5.90	LJ3a=1(R,S)2R
H37	H47	4.79	2.67	5.91	4.94	311039<br>4.88	294443<br>4.93	131594<br>5.29	190282<br>4.97	LJ3a=1(R,S)2R

* indicates two superimposed signals

Suppl Table 2

Comparison of the Measured distances on the model structures with the measured volumes and derived distances from the 100 ms NOESY spectra

Measured distance on the model structures (Å)						Measured volumes and derived distances from the 100 ms NOESY spectra				Result
Protons		1R2R	1S2R	1R2S	1S2S	LJ3a1	LJ3a2	LJ3b1	LJ3b2	
H21	H30	4.39	4.28	1.80	2.62	179675<br>4.50	∅	107604<br>3.92	103660<br>3.95	LJ3b=1(R,S)2S
H21	H31	5.21	5.14	3.27	3.70	81438<br>3.85	58925<br>4.07	135778<br>3.77	129023<br>3.80	LJ3b=1(R,S)2S
H22	H35	5.51	5.61	>6.0	>6.0	37858<br>3.90	37858<br>3.90	22293<br>3.77	22293<br>3.77	LJ3a=1(R,S)2R
H30	H46	4.89	4.87	4.00	4.08	∅	∅	17798<br>4.71	17798<br>4.71	LJ3b=1(R,S)2S
H31	H46	4.48	4.39	2.58	2.63	30726<br>4.04	30726<br>4.04	116236<br>3.45	116236<br>3.45	LJ3b=1(R,S)2S
H32	H37	4.21	4.07	4.39	4.23	73513<br>3.96	88556<br>3.80	35391<br>4.72	22142<br>5.10	LJ3a=1(R,S)2R
H33	H36	3.76	3.92	2.52	3.16	83350<br>3.84	103640<br>3.70	91508<br>4.03	129888<br>3.8	LJ3b=1(R,S)2S
H34	H37	>6.0	5.69	5.55	4.30	27434<br>4.39	34243<br>4.45	∅	20158<br>5.18	LJ3a=1(R,S)2S
H37	H46	4.68	2.36	>6.0	5.96	∅	∅	∅	∅	
H37	H47	4.79	2.67	5.91	4.94	37717<br>4.38	42793<br>4.29	∅	∅	LJ3a=1(R,S)2R

∅ no detectable signal

Supplementary methods

Full assignment of the isomers in LJ3a and LJ3b using ^{1}H NMR

To determine which sample contained the 2R and which one contains the 2S, we performed the full assignment of the isomers using ^{1}H NMR experiments (1D ^{1}H, 2D TOCSY, 2D NOESY, 2D COSY) (Suppl. Fig. S1, S2). We observed that chemical shifts of the protons involved in the racemization were different on the spectra of the two samples LJ3a and LJ3b. For example, H36/H4* was observable for one given isomer as a single correlation peak, while for the other isomer of the same sample, two correlation peaks, H36/H41 and H36/H42, were observed. In contrast, the chemical shifts of the side chains protons of the different isomers were similar in the two batches LJ3a and LJ3b and were therefore not sensitive to changes in the electronic environment of the protons attached to the carbons with the absolute configuration R or S (Figure S1B, S2B). The most important chemical shift modifications concerned the nuclei N (N6), NH (H36) and Hα (H37) of the racemic residue D1 in the two samples LJ3a and LJ3b confirming the racemization of the position P1. To identify which of the isomers (1R, 2R), (1S, 2R) on the one side and (1R, 2S), (1S, 2S) on the other side, could correspond to the recorded spectra, we built a theoretical model of each of these isomers. Then we measured on these theoretical models the characteristic inter-proton distances that discriminate the two isomers in position P2 (Suppl. Table 1, Suppl. Table 2). In a second step, we measured on the NOESY spectra, recorded at two different mixing times to account for spin diffusion artifacts, the volumes of the signals corresponding to the distances between protons measured previously on the theoretical models. The experimental distances were then determined from these volumes. Finally, knowing that the volume of the NOE between two nuclei across space depends inversely on the distance separating these two nuclei, we compared the volume of the peaks measured on the NOESY spectra, and the derived distances, to the distance determined on the theoretical models to determine which had the best match between

experimental volume and theoretical distance. Ten distances were measured on the theoretical models of the two isomers (1R, 2R), (1S, 2R) and (1R, 2S), (1S, 2S) and compared to the distances deduced from the NOE volumes. Of these 10 distances, 9 show that sample LJ3a contains the two isomers (1R, 2R) and (1S, 2R) and that sample LJ3b contains the two isomers (1R, 2S) and (1S, 2S).

Non-caspase enzyme assays

The human pro-kallikrein-1 (pro-hK1) was activated in a buffer containing: Tris-HCl 50 mM, CaCl2 10 mM, NaCl 150 mM, Brij-35 0,05% pH 7,5. Human pro-kallikrein-6 and -8 (pro-hK6 and 8 were activated using Lysyl-endopeptidase (Wako-BioProducts®) at a 1/500 weight ratio, in a buffer containing 50 mM Tris and 0,05% Brij-35 (pH 8). Kinetics using non-caspase enzymes for selectivity studies were done according to the following conditions.

**Enzymes**	**Substrates**	**Buffers**
Cathepsine B (0.2 nM)	z-RR-AMC 20 µM<br>(K_M = 169,8 µM)	Acétate de sodium 0,1 M ; EDTA 1mM ; DTT 2mM ; Brij-35 0,01 % ; pH 5,5
Cathepsine L (1.2 nM)	RLR-AMC 25 µM<br>(K_M = 13,8 µM)	Acétate de sodium 0,1 M ; EDTA 1mM ; DTT 2mM ; Brij-35 0,01 % ; pH 5,5
Cathepsine D (0.1 nM)	Mca-Gly-Lys-Pro-Ile-Leu-Phe-Phe-Arg-Leu-Lys(Dnp)-D-Arg-NH_2 10 µM	Citrate de Sodium 0,1 M ; EDTA 2 mM ; Brij-35 0,01% ; pH 4
hK1 (4 nM)	Boc-VPR-AMC 100µM	Tris 50mM; Citrate 1M; Brij-35 0.05%; pH7
hK6 (2 nM)	Boc-QAR-AMC 100 µM	Tris 50mM; Citrate 1M; Brij-35 0.05%; pH7
hK8 (1 nM)	Boc-VPR-AMC 100µM	Tris 50mM; Citrate 1M; Brij-35 0.05%; pH7
Thrombine (10 mU/mL)	Boc-VPR-AMC 100µM	Tris 50mM; Citrate 1M; Brij-35 0.05%; pH7
Plasmine (4 nM)	Boc-QAR-AMC 100µM	Tris 50mM; Citrate 1M; Brij-35 0.05%; pH7
Trypsine (0.1 nM)	Boc-QAR-AMC 100µM	Tris 50mM; Citrate 1M; Brij-35 0.05%; pH7

SUPPLEMENTARY DISCUSSION

The hydrophobic S5 site of Casp3 (hydrophobic cleft formed by two aromatic residues in the loop 4 region, Phe250 and Phe252) is similar to the S5 site in Casp2, whereas polar residues are found in equivalent positions of Casp7, suggesting that these caspases differ in their substrate selectivity at P5. Accordingly, hydrophobic P5 residues were shown to be less favorable substrates of Caspase-7 [Suppl Ref 13]. In addition, kinetics with LDESD, and LDEVD substrates showed that, Caspase-7 exhibits no preference for P5 residue in agreement with the absence of P5 interactions in the caspase-7/LDESD crystal structure (Fu et al. Apoptosis 2008) [Suppl Ref 14].

The S2 subsites of Casp7 and Casp3 are formed by identical aromatic residues (Tyr230, Trp232 and Phe282 in Casp7). Although structural models indicates that Casp7 S2 subsite can be enlarged or reduced by rotating χ angles of Tyr230, Trp232 and Phe282 to possibly accommodate larger residues [Suppl Ref 15], kinetic data have shown that Casp7 has poor cleavage efficacy of LDXXD pentapeptides containing a Proline in position P2 [Suppl Ref 16].

Taken together these structural and kinetic previous reports suggest that LJ2a and LJ3a may have a limited impact on Casp7.

SUPPLEMENTARY REFERENCES

[1] C. Griesinger, G. Otting, K. Wuthrich, and R. Ernst, “Clean Tocsy for H-1 Spin System-Identification in Macromolecules,” J. Am. Chem. Soc., vol. 110, no. 23, pp. 7870–7872. 1988.

[2] D. Marion and K. Wuthrich, “Application of Phase Sensitive Two-Dimensional Correlated Spectroscopy (cosy) for Measurements of H-1-H-1 Spin-Spin Coupling-Constants in Proteins,” Biochem. Biophys. Res. Commun., vol. 113, no. 3, pp. 967–974, 1983.

[3] M. Rance, O. Sorensen, G. Bodenhausen, G. Wagner, R. Ernst, and K. Wuthrich, “Improved Spectral Resolution in Cosy H-1-Nmr Spectra of Proteins Via Double Quantum Filtering,” Biochem. Biophys. Res. Commun., vol. 117, no. 2, pp. 479–485, 1983.

[4] J. Jeener, B. Meier, P. Bachmann, and R. Ernst, “Investigation of Exchange Processes by 2-Dimensional Nmr-Spectroscopy,” J. Chem. Phys., vol. 71, no. 11, pp. 4546–4553, 1979.

[5] A. Kumar, R. Ernst, and K. Wuthrich. “A Two-Dimensional Nuclear Overhauser

Enhancement (2d Noe) Experiment for the Elucidation of Complete Proton-Proton Cross-Relaxation Networks in Biological Macromolecules," Biochem. Biophys. Res. Commun., vol. 95, no. 1, pp. 1–6, 1980.

[6] S. Medvedeva, J. Simorre, B. Brutscher, F. Guerlesquin, and D. Marion, "Extensive H-1-Nmr Resonance Assignment of Proteins Using Natural-Abundance Gradient-Enhanced 13c-1h Correlation Spectroscopy," FEBS Lett., vol. 333, no. 3, pp. 251–256. 1993.

[7] T. Parella and J. Felix Espinosa, "Long-range proton-carbon coupling constants: NMR methods and applications," Prog. Nucl. Magn. Reson. Spectrosc., vol. 73, pp. 17–55. 2013.

[8] P. Schanda, E. Kupce, and B. Brutscher, "SOFAST-HMQC experiments for recording two-dimensional heteronuclear correlation spectra of proteins within a few seconds," J. Biomol. NMR, vol. 33, no. 4, pp. 199–211. 2005.

[9] E. F. Pettersen et al., "UCSF Chimera--a visualization system for exploratory research and analysis," J Comput Chem, vol. 25, no. 13, pp. 1605–1612. 2004.

[10] Topol, A., Tran, N. N. & Brennand, K. J. A guide to generating and using hiPSC derived NPCs for the study of neurological diseases. J Vis Exp, e52495, doi:10.3791/52495 (2015).

[11] Sun, J. et al. CRISPR/Cas9 editing of APP C-terminus attenuates beta-cleavage and promotes alpha-cleavage. Nat Commun 10, 53, doi:10.1038/s41467-018-07971-8 (2019).

[12] Cheng, C., Fass, D. M., Folz-Donahue, K., MacDonald, M. E. & Haggarty, S. J. Highly Expandable Human iPS Cell-Derived Neural Progenitor Cells (NPC) and Neurons for Central Nervous System Disease Modeling and High-Throughput Screening. *Curr Protoc Hum Genet 92:21.8.1-21.8.21.* doi:10.1002/cphg.33 (2017).

[13] Fang B, Boross PI, Tozser J, and Weber IT. Structural and kinetic analysis of caspase-3 reveals role for s5 binding site in substrate recognition. J Mol Biol. 360(3):654-666. doi: 10.1016/j.jmb.2006.05.041 (2006).

[14] Fu G, Chumanevich AA, Agniswamy J, Fang, B, Harrison RW, and Weber IT. Structural Basis for Executioner Caspase Recognition of P5 Position in Substrates. Apoptosis. 13(11): 1291–1302. (2008).

[15] Agniswamy J., Fang B., Weber IT.. Plasticity of S2-S4 specificity pockets of executioner caspase-7 revealed by structural and kinetic analysis. FEBS J. 274(18):4752-65 (2007).

[16] Julien O, Zhuang M, Wiita AP, O'Donoghue AJ, Knudsen GM, Craik CS, Wells JA. Quantitative MS-based enzymology of caspases reveals distinct protein substrate specificities, hierarchies, and cellular roles. PNAS 113(14): E2001-E2010 (2016).
