# Supplementary material for: Genuine selective caspase-2 inhibition with new irreversible small peptidomimetics
Source: Cell Death Dis. 2022 Nov 15;13(11):959. doi: 10.1038/s41419-022-05396-2 (PMC9666555; doi:10.1038/s41419-022-05396-2)
Supplement: Supplementary file 2 — Suppl. Figure S1 [file 41419_2022_5396_MOESM2_ESM.pdf]

A

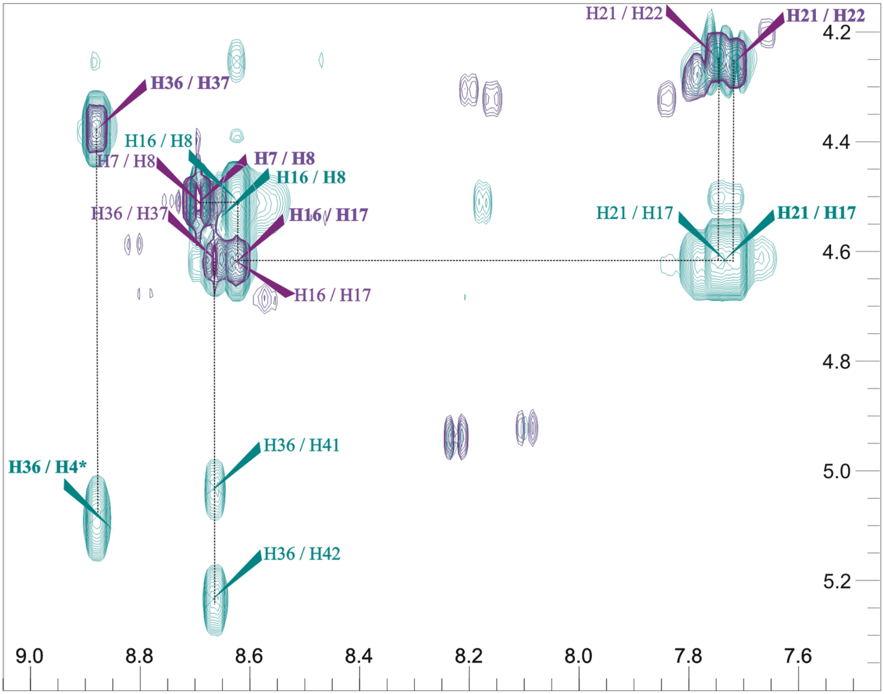

B

Residue	N	NH	αH	βH	Others
**D1**	117.07	8.88	4.38	2.49, 2.84	5.08
	118.01	8.66	4.61	2.53, 2.72	5.03, 5.23
**X2**			3.67	2.06	γCH_2: 1.48, 2.20; δCH_2: 3.47, 3.84; ε: 1.21, 1.49
			3.70	2.04	γCH_2: 1.50, 2.19; δCH_2: 3.47, 3.84, ε: 1.21, 1.49
**V3**	116.15	7.72	4.25	1.81	Hγ1# 0.76 ; Hγ2# 0.91
	116.24	7.75	4.25	1.82	Hγ1# 0.76 ; Hγ2# 0.76
**D4**	120.64	8.62	4.72	2.50, 2.65	
**V5**	107.15	8.69	4.51	2.13	Hγ1# 0.93 ; Hγ2# 0.93

Supplementary Figure S1. Bosc et al., 2021
