# Supplementary material for: Genuine selective caspase-2 inhibition with new irreversible small peptidomimetics
Source: Cell Death Dis. 2022 Nov 15;13(11):959. doi: 10.1038/s41419-022-05396-2 (PMC9666555; doi:10.1038/s41419-022-05396-2)
Supplement: Supplementary file 3 — Suppl. Figure S2 [file 41419_2022_5396_MOESM3_ESM.pdf]

A

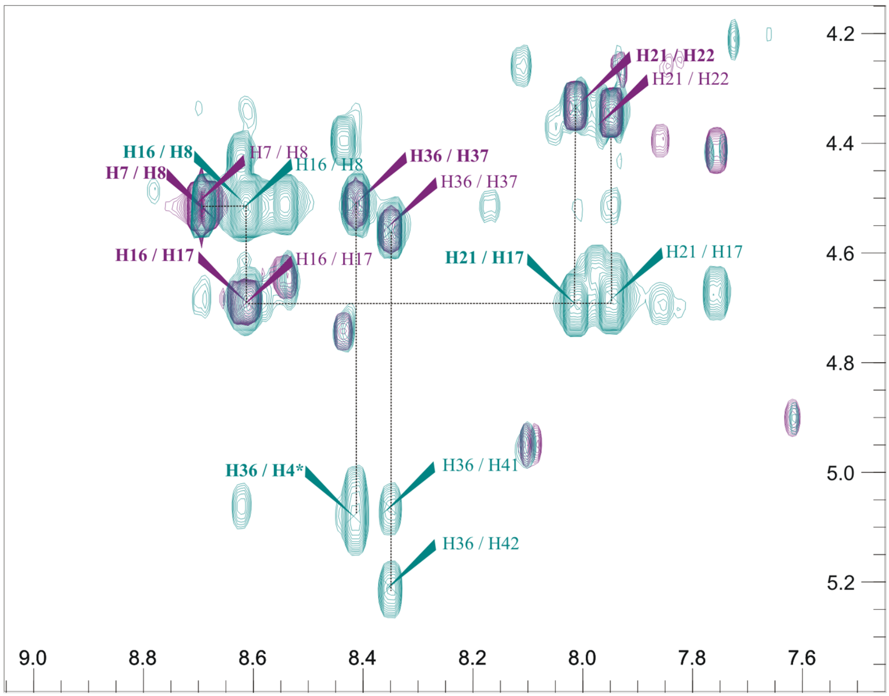

B

Residue	N	NH	αH	βH	Others
**D1**	114.2	8.41	4.51	2.49, 2.84	5.08
	113.71	8.35	4.56	2.53, 2.72	5.06, 5.21
**X2**			3.69	2.04	γCH_2: 1.49, 2.12; δCH_2: 3.50, 3.67; ε: 1.21, 1.49
			3.74	2.06	γCH_2: 1.49, 2.12; δCH_2: 3.50, 3.67; ε: 1.21, 1.49
**V3**	115.64	8.02	4.33	1.97	Hγ1# 0.79 ; Hγ2# 0.79
	115.42	7.96	4.35	1.97	Hγ1# 0.79 ; Hγ2# 0.79
**D4**	120.28	8.61	4.69	2.55, 2.69	
**V5**	106.92	8.69	4.51	2.15	Hγ1# 0.90 ; Hγ2# 0.90

Supplementary Figure S2. Bosc et al., 2021
