# Supplementary figures and images for: Genuine selective caspase-2 inhibition with new irreversible small peptidomimetics
Source: Cell Death Dis. 2022 Nov 15;13(11):959. doi: 10.1038/s41419-022-05396-2 (PMC9666555; doi:10.1038/s41419-022-05396-2)

A

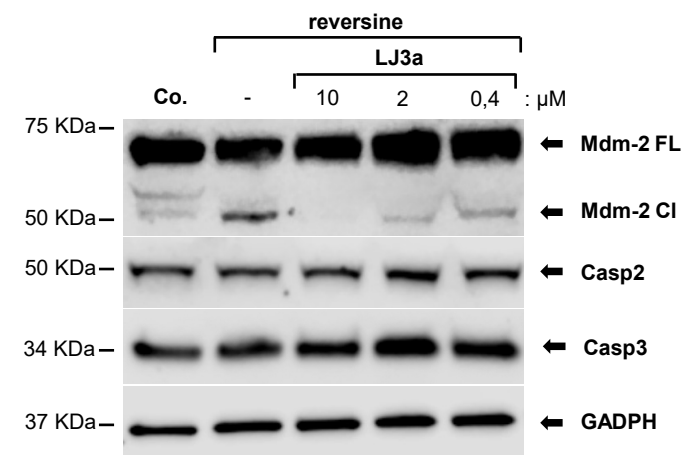

B

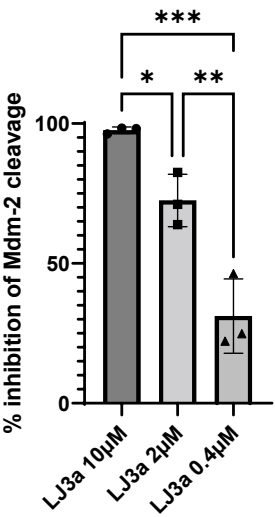

Supplementary Figure S3. Bosc et al., 2022

Supplement: Supplementary file 4 — Suppl. Figure S3 [file 41419_2022_5396_MOESM4_ESM.pdf]

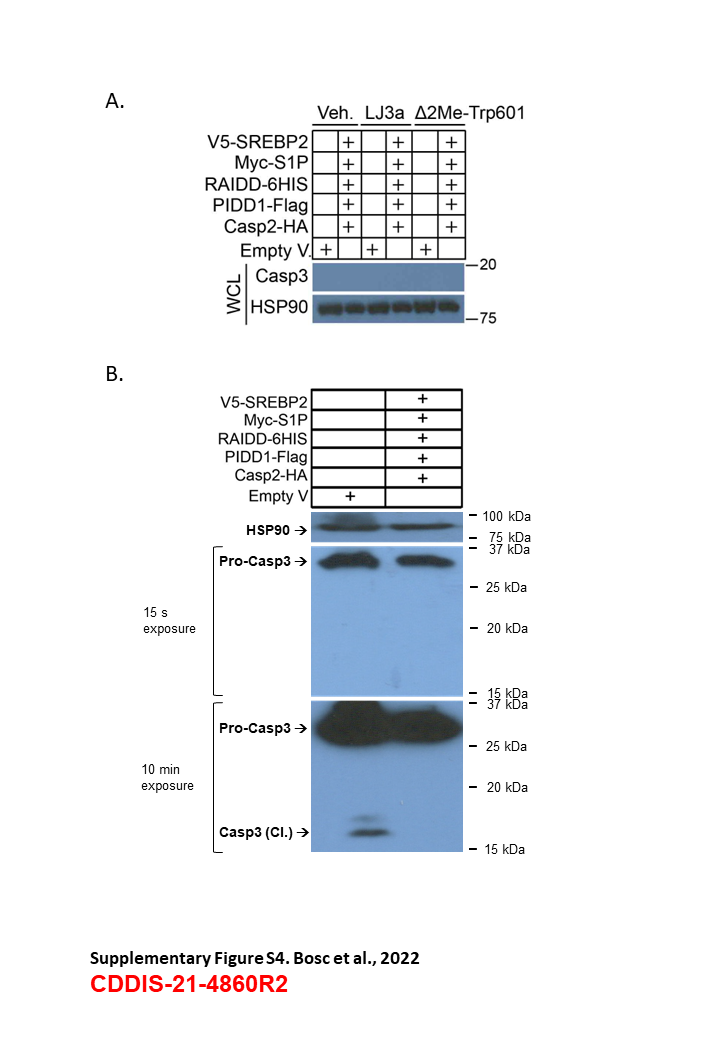

Supplement: Supplementary file 5 — Suppl. Figure S4 [file 41419_2022_5396_MOESM5_ESM.png]
