# Supplementary material for: Genuine selective caspase-2 inhibition with new irreversible small peptidomimetics
Source: Cell Death Dis. 2022 Nov 15;13(11):959. doi: 10.1038/s41419-022-05396-2 (PMC9666555; doi:10.1038/s41419-022-05396-2)
Supplement: Supplementary file 6 — Original Data File [file 41419_2022_5396_MOESM6_ESM.pdf]

Supplemental Material
Original western blots for:

Genuine Selective Caspase-2 Inhibition with new Irreversible Small Peptidomimetics

Elodie Bosc, Julie Anastasie, Feryel Soualmia, Pascale Coric, Ju Youn Kim, Lily Q. Wang, Gullen Lacin, Kaitao Zhao, Ronak Patel, Eric Duplus, Philippe Tixador, Andrew Sproul, Bernard Brugg, Michelle Reboud-Ravaux, Carol M. Troy, Michael L. Shelanski, Serge Bouaziz, Michael Karin, Chahrazade El Amri, and Etienne D. Jacotot

Fig 8b:

-Myc FL: 8bMyc

-Myc Cl.: 8b ClMyc pIRE1

-IRE1: 8b ClMyc pIRE1

-HDAC: 8bHDAC

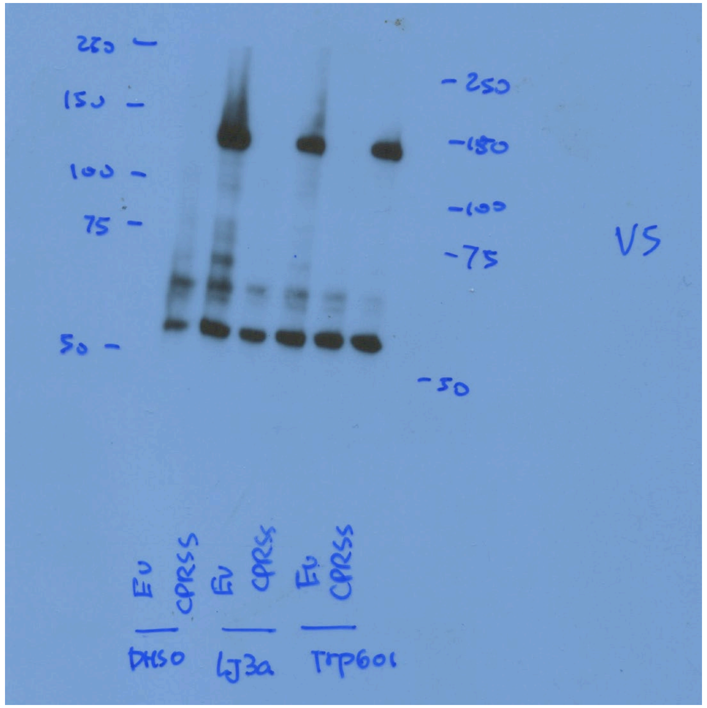

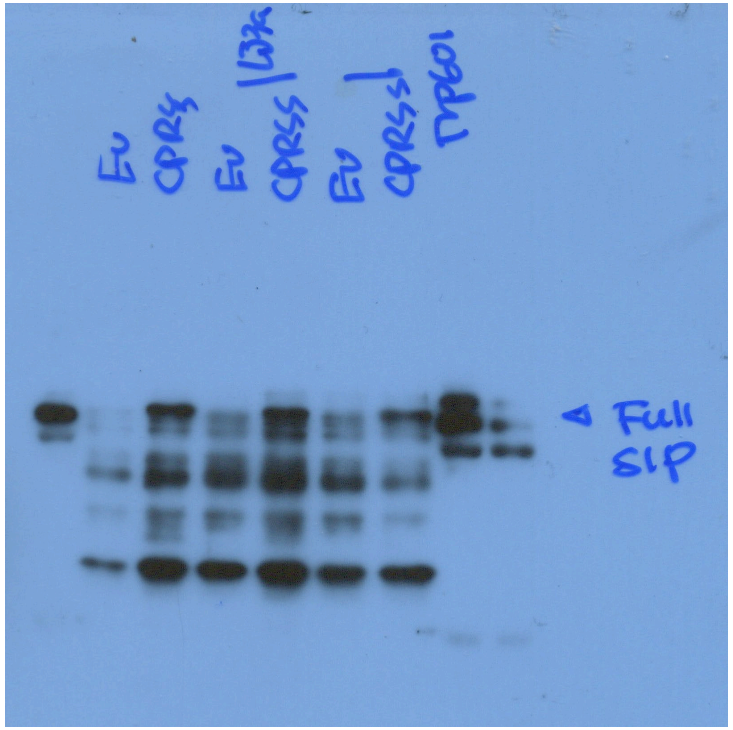

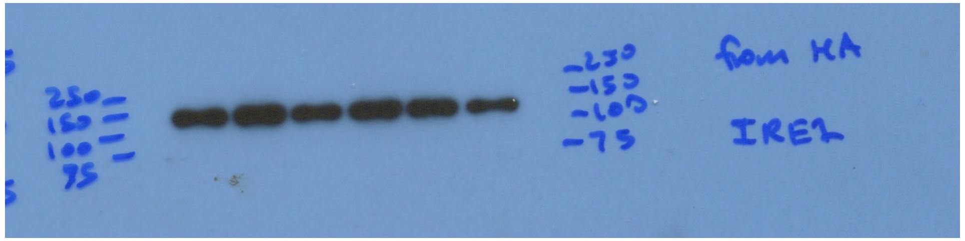

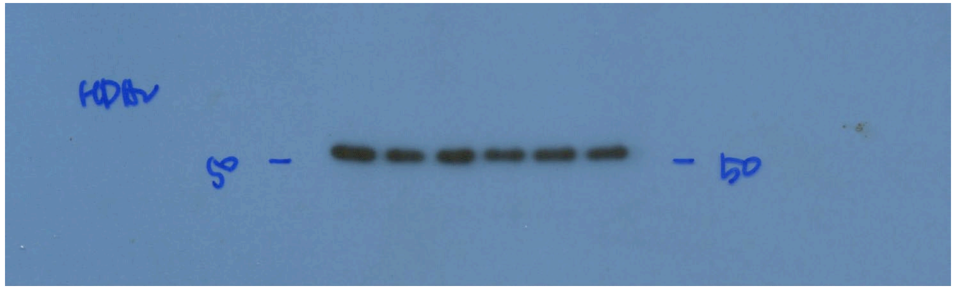

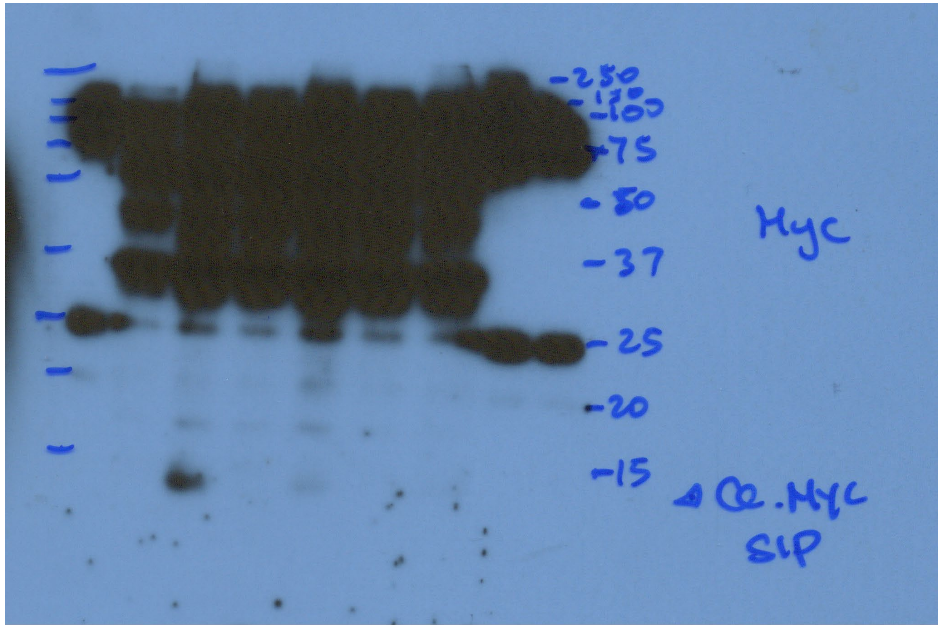

Fig 8c

-V5 Fl: 8cV5

-V5 Cl.: 8cV5Cl

-Tubulin: 8c Tub

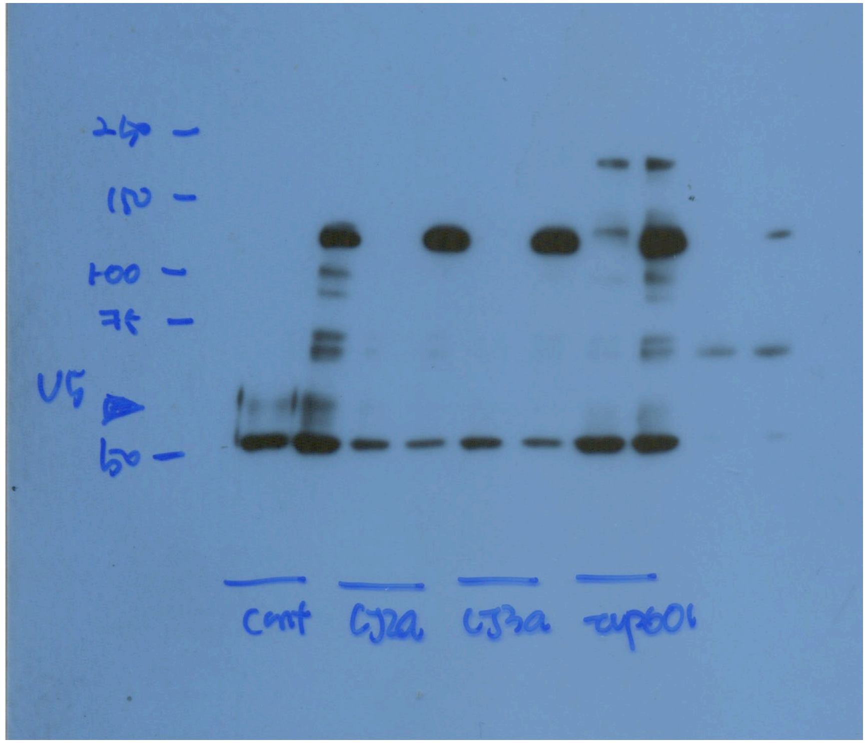

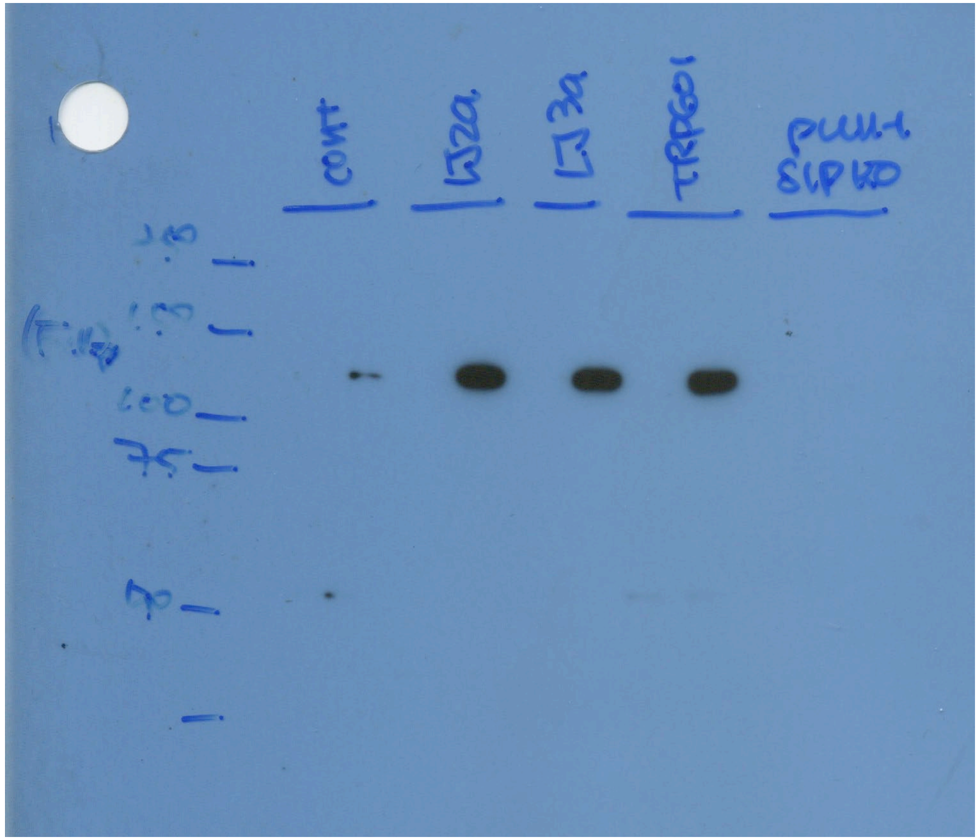

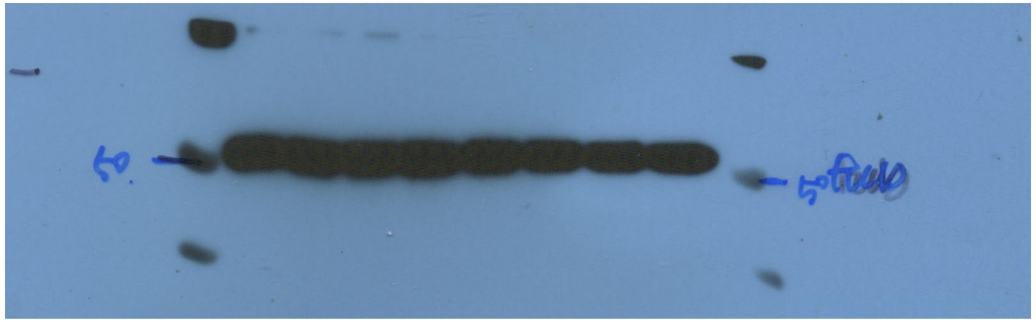

Fig 8d:

-HA: 8d HA
-Flag: 8d Flag His
-6HIS: 8d Flag His
-HSP90: 8dHSP

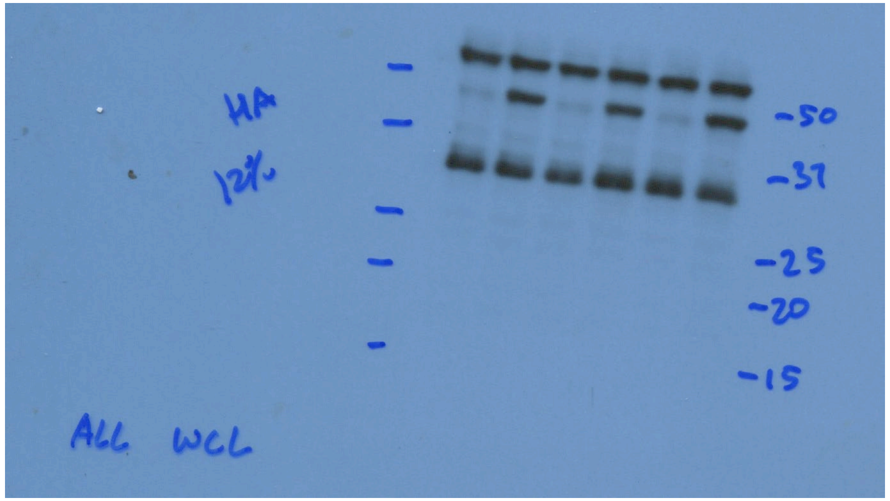

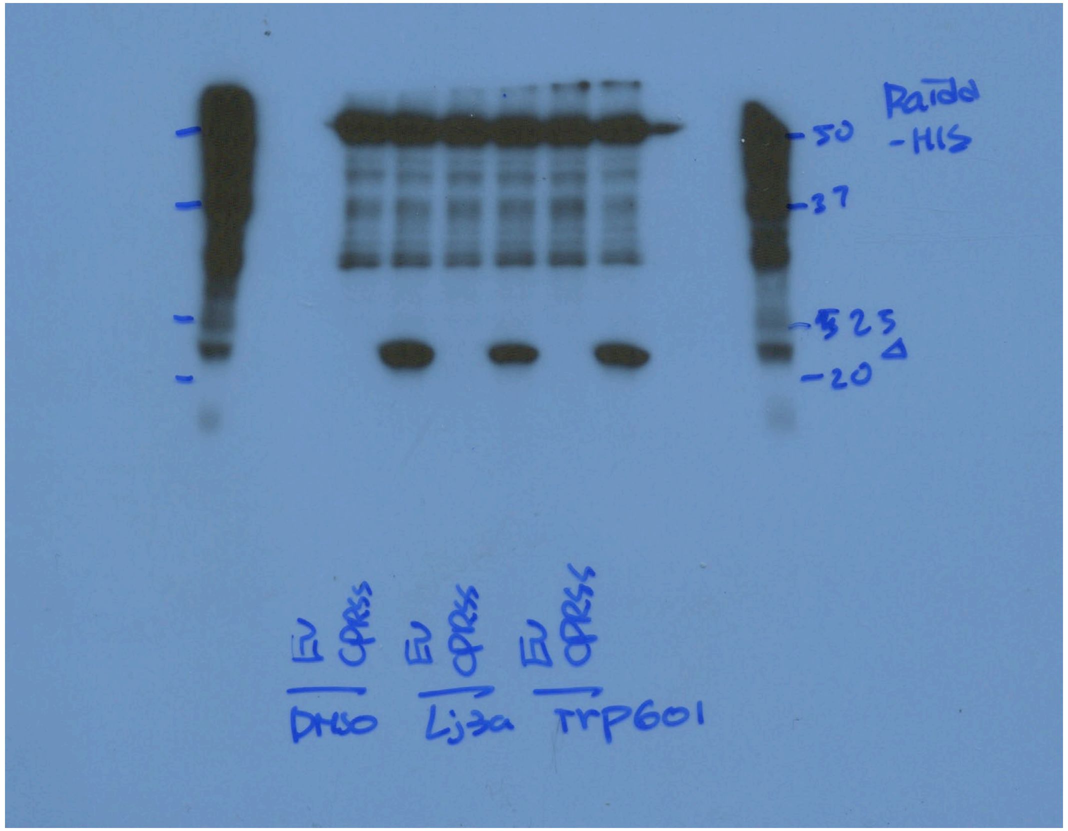

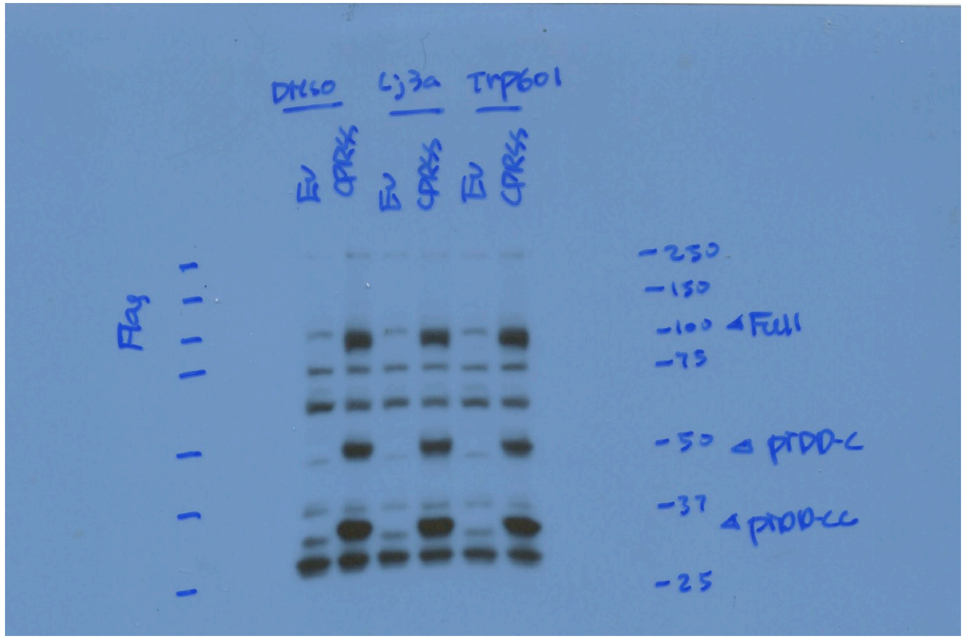

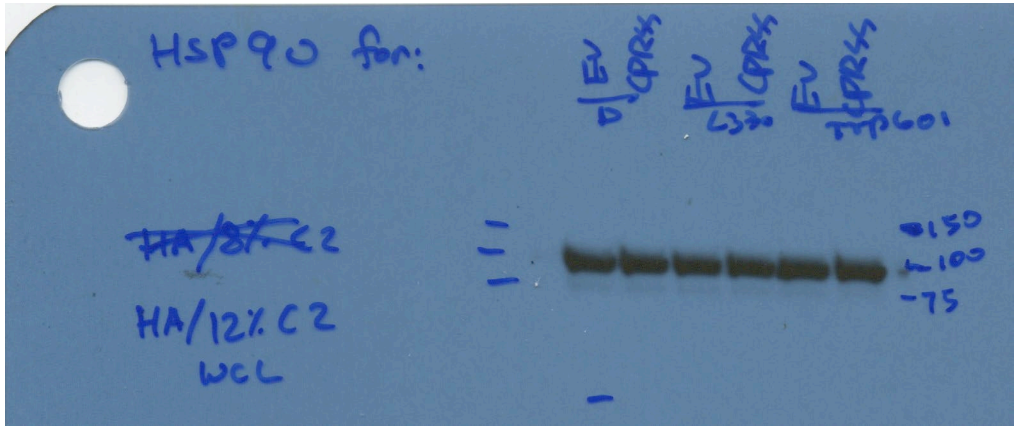

Supp. Fig S4A:
-Casp3: S4A C3
-HSP90: 8dHSP

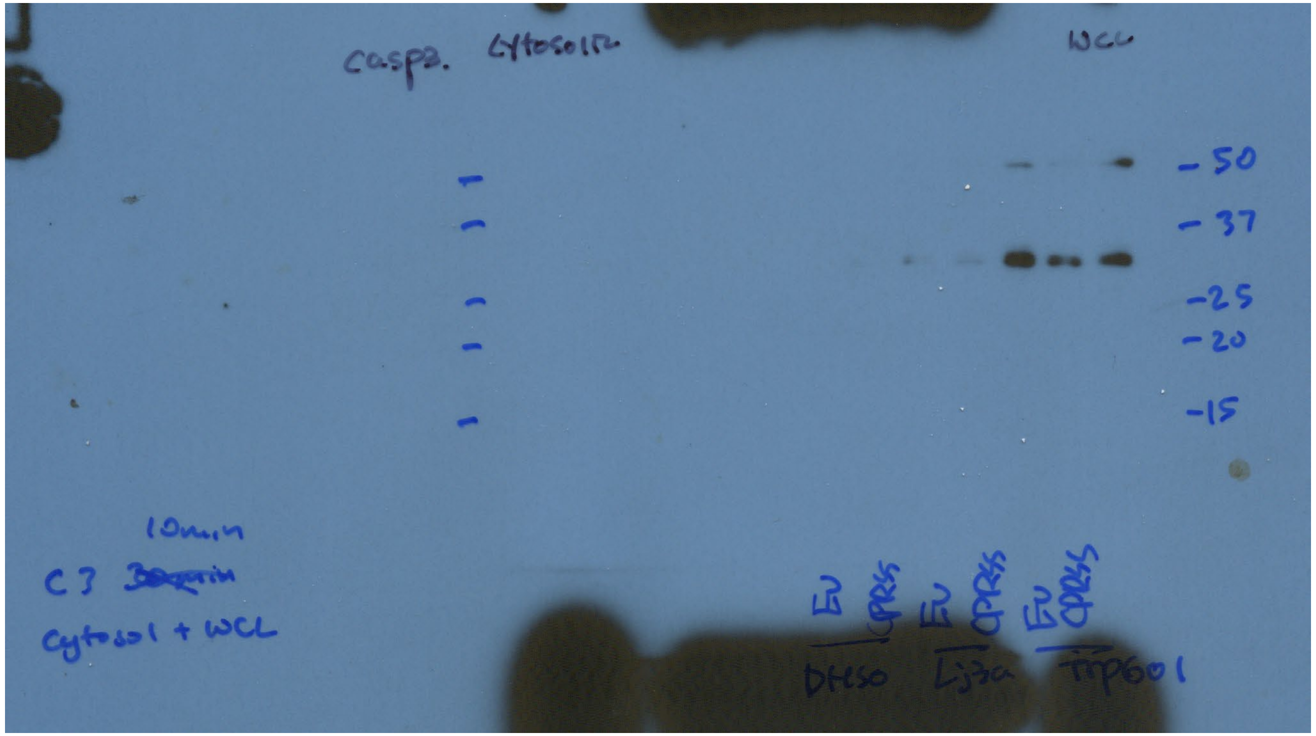

Casp3

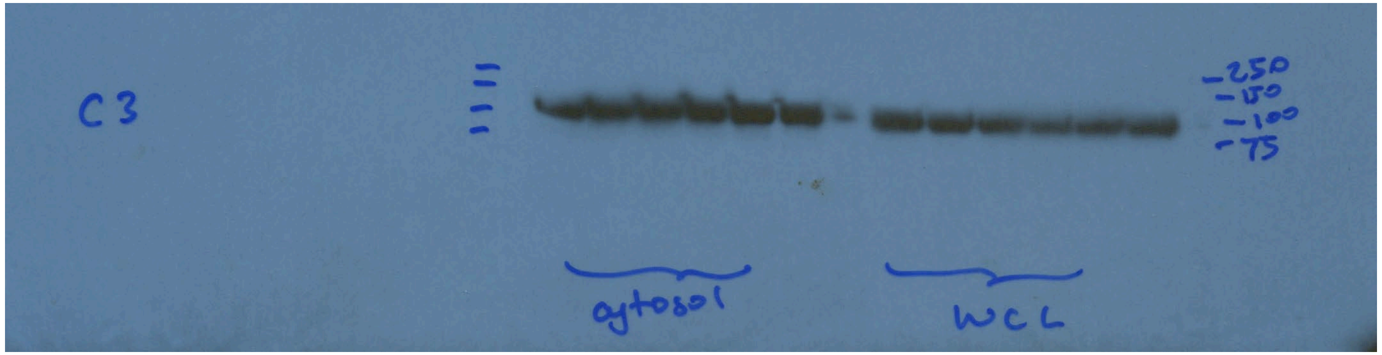

HSP90

Supp. Fig S4B:

-HSP90: HSP 3rd

-Pro-C3: C3 15s exposure

-Pro-C3 + C3 (Cl.): C3c 10min exposure

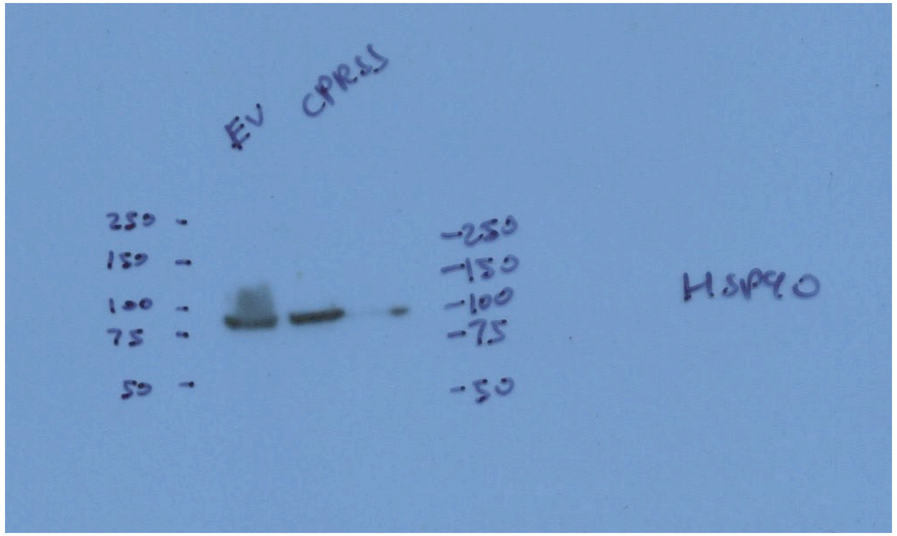

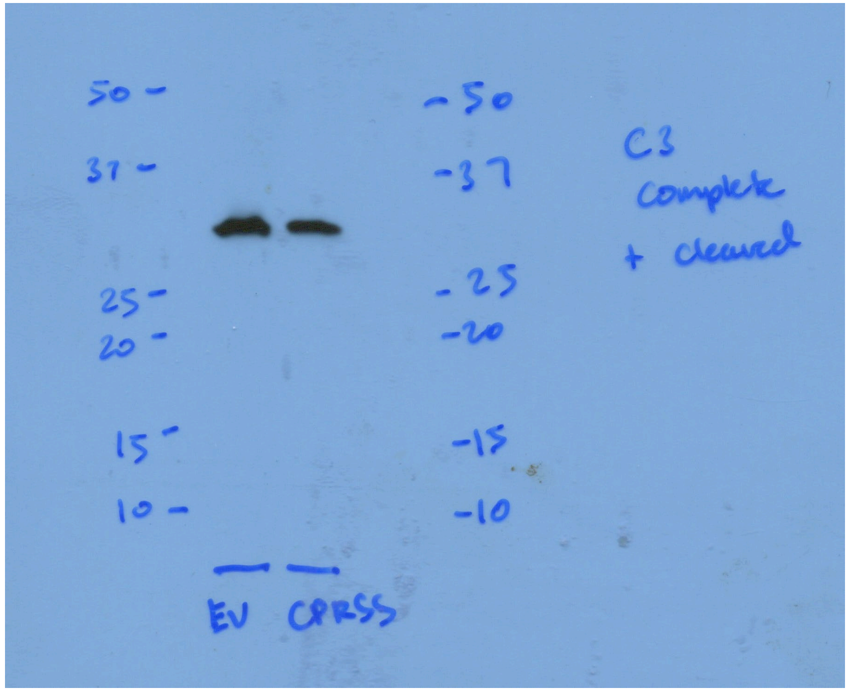

15 sec. exposure

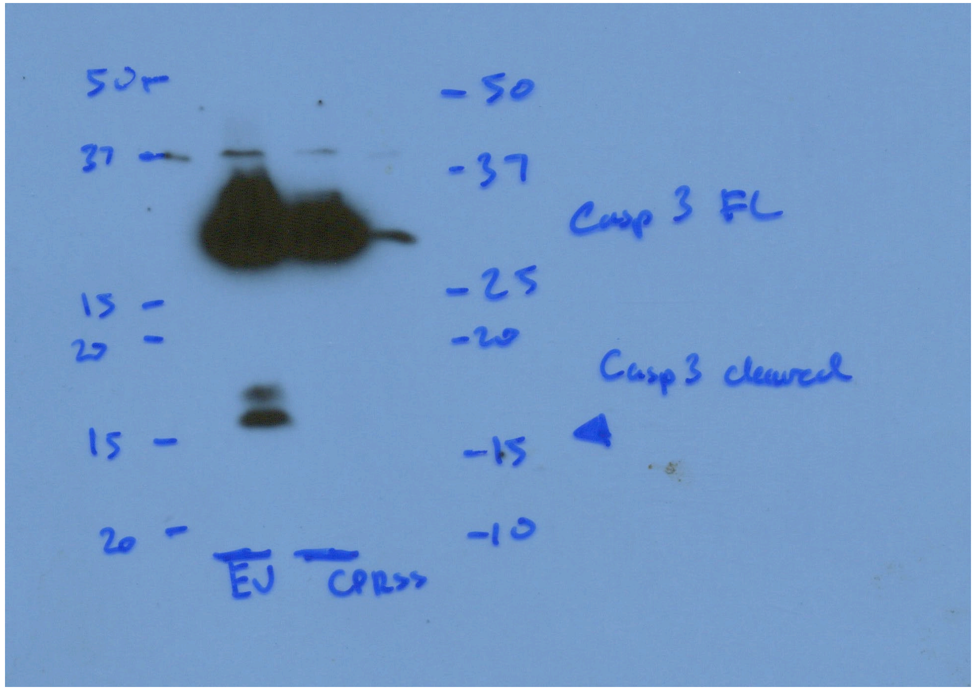

10 min. exposure
