# Supplementary material for: Genuine selective caspase-2 inhibition with new irreversible small peptidomimetics
Source: Cell Death Dis. 2022 Nov 15;13(11):959. doi: 10.1038/s41419-022-05396-2 (PMC9666555; doi:10.1038/s41419-022-05396-2)
Supplement: Supplementary file 7 — Suppl. Table 1 [file 41419_2022_5396_MOESM7_ESM.pdf]

Bosc et al. Supplementary Table 1

Comparison of the Measured distances on the model structures with the measured volumes and derived distances from the 400 ms NOESY spectra.

Measured distance on the model structures (Å)						Measured volumes and derived distances on 400 ms NOESY spectra				Result
Protons		1R2R	1S2R	1R2S	1S2S	LJ3a1	LJ3a2	LJ3b1	LJ3b2	
H21	H30	4.39	4.28	1.80	2.62	550018<br>4.43	*	820696<br>3.90	693644<br>4.00	LJ3b=1(R,S)2S
H21	H31	5.21	5.14	3.27	3.70	294592<br>4.92	354974<br>4.77	1050000<br>3.74	976086<br>3.74	LJ3b=1(R,S)2S
H22	H35	5.51	5.61	>6.0	>6.0	481831<br>4.04	481831<br>4.04	320176<br>4.06	320176<br>4.06	LJ3a=1(R,S)2R
H30	H46	4.89	4.87	4.00	4.08	110876<br>5.16	110876<br>5.16	96120<br>4.96	96120<br>4.96	LJ3b=1(R,S)2S
H31	H46	4.48	4.39	2.58	2.63	214238<br>4.63	214238<br>4.63	800000<br>3.49	800000<br>3.49	LJ3b=1(R,S)2S
H32	H37	4.21	4.07	4.39	4.23	520824<br>4.48	656653<br>4.31	346066<br>4.50	470582<br>4.28	LJ3a=1(R,S)2R
H33	H36	3.76	3.92	2.52	3.16	554349<br>4.43	801356<br>4.17	801016<br>3.91	1010000<br>3.76	LJ3b=1(R,S)2S
H34	H37	>6.0	5.69	5.55	4.30	239313<br>5.00	231267<br>5.13	113630<br>5.00	100462<br>5.50	LJ3a=1(R,S)2S
H37	H46	4.68	2.36	>6.0	5.96	89113<br>6.00	94410<br>5.96	65388<br>5.94	67752<br>5.90	LJ3a=1(R,S)2R
H37	H47	4.79	2.67	5.91	4.94	311039<br>4.88	294443<br>4.93	131594<br>5.29	190282<br>4.97	LJ3a=1(R,S)2R

* indicates two superimposed signals
